# Spirometric Pulmonary Restriction in Herbicide-Exposed U.S. Vietnam War Veterans

**DOI:** 10.3390/ijerph16173131

**Published:** 2019-08-28

**Authors:** Yasmin Cypel, Stella E. Hines, Victoria J. Davey, Stephanie M. Eber, Aaron I. Schneiderman

**Affiliations:** 1 Epidemiology Program, Post Deployment Health Services (10P4Q), Office of Patient Care Services, Department of Veterans Affairs, Veterans Health Administration, 810 Vermont Avenue, NW, Washington, DC 20420, USA; 2 Department of Medicine, Baltimore Veterans Affairs Medical Center, Baltimore, MD 21201, USA; 3 Division of Occupational and Environmental Medicine and Division of Pulmonary & Critical Care Medicine, Department of Medicine, School of Medicine, University of Maryland-Baltimore, Baltimore, MD 21201, USA; 4 Office of Research & Development (10X2), Veterans Health Administration, Department of Veterans Affairs, Washington, DC 20420, USA

**Keywords:** Herbicides, veterans, restrictive pulmonary disease, Vietnam War, chemical exposures, spirometry

## Abstract

Spirometric restriction in herbicide-exposed U.S. Army Chemical Corps Vietnam War veterans was examined because no published research on this topic in Vietnam War veterans exists. Spirometry was conducted on 468 veterans who served in chemical operations in a 2013 study assessing the association between chronic obstructive pulmonary disease (COPD) and herbicide exposure. Exposure was verified based on blood serum values of 2,3,7,8-tetrachlorodibenzo-p-dioxin. Further, the association between herbicide exposure and spirometry restriction (forced expiratory volume in one second (FEV_1_)/forced vital capacity (FVC) ≥ lower limit of normal (LLN) and FVC < LLN) was tested after adjustment for military characteristics, selected anthropometrics, and other predictors using multivariable regression. Spirometric restriction in herbicide sprayers (15.7%, 95% CI: 10.6, 20.9) was almost twice that of nonsprayers (9.91%, 95% CI: 5.9, 13.9) (*p* = 0.081). While spirometric restriction was not significantly associated with herbicide exposure (adjusted odds ratio (aOR) = 1.64, 95% CI: 0.82, 3.29) despite the greater prevalence of restriction in sprayers versus nonsprayers, spirometric restriction was significantly associated with race/ethnicity (aOR = 3.04, 95% CI: 1.36, 6.79) and waist circumference (aOR = 2.46, 95% CI: 1.25, 4.85). Because restrictive pulmonary disease may result from chemically-induced inflammation or sensitivity, research on chemical exposures and restriction in veterans should continue. Future study should include full pulmonary function testing, targeted research designs, and a wider set of explanatory variables in analysis, such as other determinants of health.

## 1. Introduction

The scientific literature on the associations between herbicide exposure and chronic disease in Vietnam Era veterans (those who served in Vietnam and in other areas during the War) has been examined every two years by the National Academy of Sciences (NAS) as required by congressional mandate. The result is *Veterans and Agent Orange* reports, a published series that was first released in 1994 [1]. These reports represent a comprehensive review of the scientific literature addressing the chemicals of interest with studies that span epidemiological, environmental, occupational, and toxicological research. Through its most recent review, NAS has indicated that there are no conclusive findings about the relationship between these chemicals and non-cancerous respiratory diseases [2]. The NAS classified the latter as either an airway disease, which is primarily characterized by asthma and chronic obstructive pulmonary disease (COPD) where the airflow out of the lungs is affected, or parenchymal or restrictive disease where the inflammation and scarring of the deep lung tissue may be present. The physiologic processes in restrictive lung diseases lead to decreases in lung volumes, which are confirmed with lung volume measurement by inert gas or body plethysmographic methods. Certain patterns on spirometric testing, however, can suggest the presence of a restrictive process and spirometric endpoints have been used to assess responses to medical treatment of interstitial lung diseases [3,4]. 

Restrictive lung disease includes interstitial lung diseases (such as various forms of pulmonary fibrosis, sarcoidosis and pneumoconiosis), impacts from morphology including obesity or chest wall mass/constriction, or neuromuscular weakness which occurs in amyotrophic lateral sclerosis. Interstitial lung diseases can arise because of the sensitivity to or inflammation from herbicides (e.g., paraquat and pulmonary fibrosis in animal studies) [5] or similar types of chemicals like pesticides (e.g., hypersensitivity pneumonitis) [6,7]. Restrictive pulmonary disease is associated, in general populations, with an increased risk of mortality and comorbidities [8,9,10,11,12,13,14], like metabolic syndrome and cardiovascular disease. 

Past research has provided findings on non-cancerous respiratory diseases for Vietnam War veterans [15,16,17,18,19,20,21,22,23,24,25,26,27,28]. Some studies have reported on COPD or other specific pulmonary disorders [18,19,23,24,25,26,27,28], but there are no published studies on restrictive lung diseases in Vietnam War veterans. Given this and concerns over the associations between respiratory disease and herbicide exposure, particularly among members of the U.S. Army Chemical Corps (ACC) who served in chemical operations during the Vietnam War, a deeper examination of the associations between restrictive pulmonary physiology and herbicide exposure deserves further study. 

The purpose of this paper is to examine the relationship between herbicide exposure and spirometric restriction in ACC veterans. Secondarily, this study examined the association between spirometric restriction and other covariates to determine their relevance as potential risk factors.

## 2. Materials and Methods 

**Figure 1:**
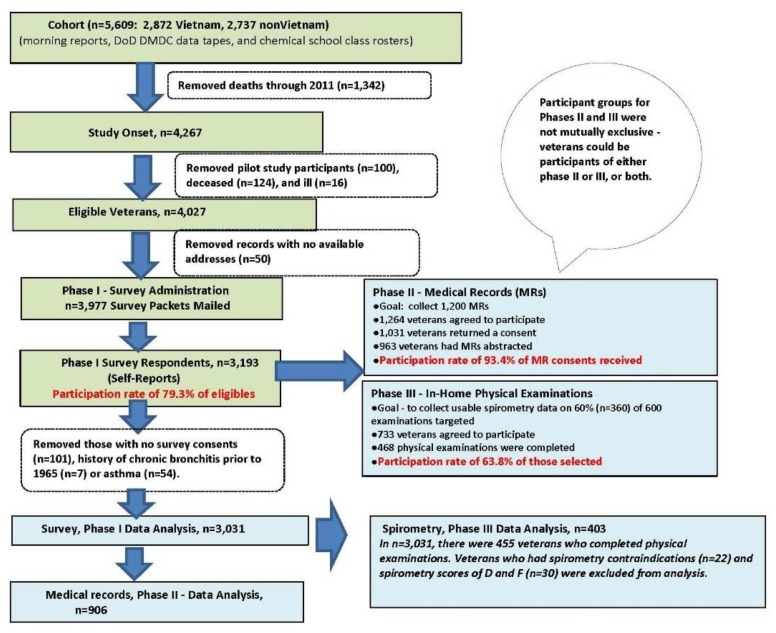
Data collection, design, and analysis.

The data evaluated here come from a subset of veterans who participated in a larger study, the 2013 Army Chemical Corps Vietnam-Era Veterans Health Study. That study included three different phases: A health survey (phase I), a medical records review (phase II), and a physical examination (phase III) conducted in veterans’ homes that included anthropometrics and spirometry (Figure 1). The main objective of the study was to assess hypertension and COPD in relation to herbicide exposure using the health survey, while the phase II and III subanalyses were conducted to assess the self-reported survey data. For phase III, the analyses were constrained to the parameters provided only by spirometry because no data were collected on the full pulmonary function tests (e.g., assessment of total lung capacity, functional residual capacity or residual volumes) or other more advanced clinical pulmonary tests. The additional details of the study’s three phases and other design considerations have been presented elsewhere [26,29].

In phase III, 733 survey participants agreed to complete a physical examination and 468 completed it (63.8% response rate) (Figure 1). Of these, after accounting for spirometry contraindications and unusable data, 403 veterans completed spirometry. Thirty poor quality scores were identified where only 1 or fewer spirometry trials were deemed acceptable. An acceptable spirometry maneuver was classified as being free from errors, such as a poor initial blast or exhalation for less than 6 seconds or the lack of plateau, as per American Thoracic Society/European Respiratory Society (ATS/ERS) recommendations [30]. Spirometry was considered repeatable when the highest and second highest acceptable values for forced vital capacity (FVC) and forced expiratory volume in one second (FEV_1_) were within 150 milliliters of each other. 

All subjects whose data were used in this analysis gave their informed consent for study inclusion. The U.S. Department of Veterans Affairs’ (VA) guidelines on the public use of data are not finalized. The data from this study are not currently available for external use. However, VA supports efforts to provide limited, restricted access to research data under written agreements consistent with commitments made to protecting the privacy and confidentiality of research participants and subject to resource availability. The study and informed consent were performed in accordance with the guidelines on the protection of human subjects of research laid forth by the Belmont Report. The protocol (MIRB# 01359) was approved by the Washington DC VA Medical Center’s Institutional Review Board.

### 2.1. Pulmonary Function Measures

Spirometry was performed using EasyOne^TM^ diagnostic spirometers (Model 2001, ndd Medical Technologies, Inc., Andover, Massachusetts, USA) following ATS/ERS guidelines [30]. Eighteen technicians, who administered spirometry for veterans residing across the U.S., were trained on spirometry by staff from the National Institute for Occupational Safety and Health (NIOSH). The veterans were spirometry ineligible if they screened positive for any of 15 contraindications (e.g., use of inhaled medication 24 h prior to testing, current illness). NIOSH reviewed each session’s spirometry flow curves for technical quality and reviewed spirometer-derived quality grades (A–D, F grades) based on their repeatability and acceptability rules. The spirometry results with grades A–C were used in the analysis [31,32,33,34]. The ATS/ERS repeatability criteria were met for grades A–B. Grade C tests met acceptability standards, but not strict repeatability criteria. These tests comprised 14% of the 403 usable records and were included in the analysis according to ATS/ERS [30] and NIOSH [33] guidance. The spirometry output was compared to the reference values for males based on age, height, and race/ethnicity data obtained from the National Health and Nutrition Examination Survey (NHANES) III [35].

Spirometric restriction was based on the following pulmonary parameters: FEV_1_, FVC, FEV_1_/FVC ratio, and LLN (lower limit of normal for a specified parameter is the lower 5th percentile of the normal value for a healthy, nonsmoking population by age, height, sex, and race/ethnicity [35]), and defined as FEV_1_/FVC ≥ LLN and FVC < LLN [3,35]. The means and standard deviations for the percentage of the predicted FEV_1_ and FVC and prevalence with its 95% confidence interval (CI) stratified by exposure status (herbicide spraying versus no spraying) are reported. A mixed process of spirometric restriction and obstruction was reported and defined as FEV_1_/FVC < LLN & FVC < LLN. 

### 2.2. Other Measures

Most variables used in the regression analysis came from the data collected during the in-home physical examinations. These included age at the time of the study (years; 59–64 (referent group), 65–69, 70 and over), race/ethnicity (white (referent group), black, other nonwhite), body mass index (BMI, kilograms/meters^2^ (kg/m^2^), calculated from measured height and weight: ≤24.9 (normal/underweight) (referent group), 25–29.9 (overweight), ≥30 (obese)), waist circumference (for men: ≥102 centimeters (cm), <102 cm (referent group)) [36], waist: hip ratio (for men: ≥0.90, <0.90 (referent group)) [37], measured height (cm) and the veterans’ spirometric restriction status (spirometric restriction versus no spirometric restriction (referent group)). 

The variables obtained from the survey included self-reports of herbicide exposure (herbicide spray status represented as sprayer versus nonsprayer (referent group); “Now thinking about herbicides, did you yourself ever mix, handle, or spray these chemicals while you were in the military?”); physician diagnosed COPD (COPD or no COPD (referent group)) and hypertension (hypertension or no hypertension (referent group)); and cigarette smoking status (current smoker, former smoker, nonsmoker (referent group)). The diabetes data (diabetes versus no diabetes (referent group)) were obtained from medical records. Hypertension and diabetes were initially included in our analysis because these conditions were associated with spirometric restrictive disease in nonveteran populations [8,10,11,13,38]. Hypertension was also viewed as a potential confounder because it has been significantly associated with herbicide exposure (adjusted odds ratio (aOR) = 1.74; 95% confidence interval (CI): 1.44, 2.11) in ACC veterans [29]. This was similarly the case for diabetes (aOR = 1.50, 95% CI: 1.15, 1.95) [17]. Mortality risk also has been shown to be elevated with spirometric restriction [8,9,12,14]. Self-reported COPD was included in the analysis to control for potential misdiagnosis of lung disease. 

This study used herbicide spray status as an indicator for herbicide exposure because past findings showed statistically significant differences (*p* ≤ 0.05) in blood serum dioxin levels (namely, 2,3,7,8-tetrachlorodibenzo-p-dioxin (TCDD)) between veterans who served in Vietnam and sprayed herbicides versus either those who served in Vietnam but did not spray or those who did not serve in Vietnam with no spray history [17,29]. Serum TCDD was evaluated because it is a toxic contaminant of Agent Orange and other herbicides which have been associated with chronic health conditions in Vietnam War veteran and nonveteran populations [1,2]. Serum dioxin determination, however, could not be made for the participants in the 2013 ACC study because of the amount of time that had elapsed since exposure given dioxin’s 7–11-year half-life [39,40]. Further reasons for this study’s reliance on self-reported exposure is because there were no measurements of individual-level herbicide exposure during the Vietnam War. 

The data for service variables were obtained from military personnel records collected from the National Personnel Records Center (U.S. National Archives and Records Administration, St. Louis, Missouri), the morning reports of ACC units stationed in Vietnam, Defense Manpower Data Center tapes of Vietnam Era Army active duty personnel, and class rosters from the Army Chemical School (Ft. McClellan, Alabama) [20]. A variable called Vietnam service status was created that was a dichotomous variable representing two cohorts of veterans—one cohort of men who served in-theater in Vietnam (hereafter, Vietnam) versus a second cohort of men who never served in Southeast Asia (primarily Europe, U.S.) (hereafter, non-Vietnam). Vietnam service status (Vietnam, non-Vietnam (referent group)), military rank (enlisted, officer (referent group)), and military duration (18–23 months (mos) (referent group), 24–36 mos, > 36 mos) were analyzed. 

### 2.3. Statistical Analysis

SAS/STAT^®^ software (Version 9.4, SAS Institute Inc., North Carolina, USA) [41] was used for all analyses. Descriptive statistics were reported. The differences in the means and percentages were determined using t-tests and risk (proportion) difference tests, respectively. Ninety-five percent confidence intervals (CIs) were generated for continuous variables and for the prevalence of spirometric restriction. Bivariate, unadjusted associations were assessed through Pearson chi-square tests using either cross-tabulation or logistic regression. The analyses were unweighted.

Correlation matrices were computed to evaluate multicollinearity using Pearson’s product moment and Spearman rank order correlation coefficients. The multivariable logistic regression analysis was used to evaluate first-order interactions, the main effects derived from the association between spirometric restriction and herbicide exposure after adjusting for other independent variables, as well as the association between spirometric restriction with other predictors. The original set of independent variables examined was: Age, race/ethnicity, herbicide spray status, Vietnam service status, military service duration, military rank, cigarette smoking status, hypertension, diabetes, COPD, height, BMI, waist circumference, and waist: hip ratio. A hierarchical approach helped to determine what predictors should remain in the model using the goodness-of-fit tests (e.g., Akaike Information Criterion, Hosmer-Lemeshow) and other indicators [42]. This approach involved the sequential addition of demographics, military, and then health-based variables into the regression with the evaluation of their impact on the resultant regression output (not shown in tables). The conceptual relevance of predictors, like height and Vietnam service status for example, was also considered when determining the final variable inclusion. 

BMI, waist: hip ratio, and diabetes were dropped in regression because they were not statistically significant; their addition to the model did not change the overall association between spirometric restriction and exposure. BMI and waist: hip ratio were highly correlated with waist circumference; and dropping BMI resulted in little change in Wald chi-square test statistics (not shown in tables) or aORs for the remaining model variables. Visceral adiposity, as measured by waist circumference, may also be more highly associated with spirometric restriction than BMI [12], and unlike BMI and waist: hip ratio, the adjusted association between waist circumference and spirometric restriction remained elevated and statistically significant despite model variations. COPD, although no longer significant after adjustment, was retained because of the potential for misclassification regarding clinical diagnosis of COPD and spirometric restriction, or for insights into the likelihood of mixed disease types. Hypertension was dropped because its effect was not statistically significant. The final model included the following independent variables: Age, race/ethnicity, herbicide spray status, Vietnam service status, military service duration, rank, waist circumference, cigarette smoking status, height, and COPD. 

The aORs and their associated 95% CIs are reported and were derived from a single multivariable model. The global null hypothesis (likelihood ratio (LR) chi-square test) result for the final model is reported. Two-tailed statistical tests were conducted and *p* values ≤ 0.05 were deemed statistically significant. The study was powered based on an earlier, main portion of the study that focused specifically on hypertension and COPD, where both the prevalence of self-reported hypertension and COPD were examined [26,29].

## 3. Results

Table 1 shows the characteristics of the participants stratified by herbicide exposure. The characteristics that were significantly related to herbicide exposure were age (years, continuous, *p* = 0.053), whether a veteran served in Vietnam (*p* < 0.0001), the time served in the military (*p* = 0.017), and self-reported physician diagnosed hypertension (*p* = 0.011) and COPD status (*p* < 0.0001). Most participants were between 65 and 69 years of age, but sprayers were more likely to be ≥70 years old (19.4%) than nonsprayers (12.7%). Most participants were white (74.7%), served in-theater (67.5%), enlisted (84.6%), and served 2–3 years (54.1%). Most were former cigarette smokers (52.4%), were overweight or heavier (83.7%), had waist: hip ratios greater than or equal to 0.90 (84.8%), and had waist circumference greater than or equal to 102 centimeters (46.9%). There was no significant difference in height by herbicide spray status (*p* = 0.89). Nearly 20% of all veterans reported a diabetes diagnosis, 71.9% reported being diagnosed with hypertension, and 16.4% reported a diagnosis of COPD. From the 403 veterans, 47.4% were herbicide sprayers, while 52.6% were nonsprayers.

Table 2 shows the spirometric characteristics and spirometric restriction prevalence of these veterans. There were no significant differences for the following: FEV_1_, FVC, FEV_1_/FVC, and percentage of predicted for FEV_1_ and FVC, respectively. The mean FVC (± standard deviation) for exposed veterans was 3.90 (± 0.78), which did not differ from the FVC mean estimate (4.04 (± 0.83)) obtained for unexposed veterans (*p* = 0.082). Although the prevalence of spirometric restriction was not significantly different (*p* = 0.08) between sprayers and nonsprayers, the percentage of sprayers with spirometric restriction was almost twice that of nonsprayers (15.7% (95% CI: 10.6, 20.9), sprayers; 9.91% (95% CI: 5.9, 13.9), nonsprayers). Eighteen veterans, or 4.5% (95% CI: 2.8, 7.0), met the criteria for a mixed process of spirometric restriction and obstruction. There was no difference between sprayers (4.7%, 95% CI: 1.7, 7.7) and nonsprayers (4.3%, 95% CI: 1.5, 7.0) on the prevalence of a mixed process of spirometric restriction and obstruction (*p* = 0.82). 

Table 3 shows the aORs and 95% CIs representing the association between spirometric restriction and each predictor. The variables found to be statistically significant in unadjusted cross-tabulations, such as self-reported physician diagnosed COPD and Vietnam service status, were no longer significant after adjustment using multivariable logistic regression. The association between whether a veteran had spirometric restriction and the total set of predictors was significant (LR x^2^= 27.74, 14 df, *p* = 0.015) suggesting that at least one of the regression coefficients was statistically significant. The association between herbicide exposure and spirometric restriction was not significant (aOR = 1.64, 95% CI: 0.82, 3.29) after adjustment for all remaining independent variables. Race/ethnicity and waist circumference were each significantly associated with spirometric restriction after controlling for other independent variables. The estimated odds of developing spirometric restriction in black veterans were 3.0 times the odds for white veterans after adjusting for height, smoking, and other covariates (aOR = 3.04, 95% CI: 1.36, 6.79). For waist circumference, the odds of spirometric restriction among those with waist circumference greater than or equal to 102 cm were 2.5 times the odds for veterans with waist circumference less than 102 cm (aOR = 2.46, 95% CI: 1.25, 4.85).

## 4. Discussion

This analysis of the data from the 2013 ACC Vietnam-Era Veterans Health Study is important because it is the only study to date of spirometric restriction in Vietnam War veterans. Overall, the prevalence of spirometric restriction in ACC herbicide-exposed veterans did not significantly differ from the unexposed despite the results that demonstrated a much higher proportion of this condition among sprayers. The authors believe that this could be partly based on anthropometric and racial differences, but social health determinants related to health services access, disease susceptibility, or social and economic disadvantage [43] could be other important reasons underlying the differences. These factors could not be examined because these data were not collected.

The significant and independent association between race/ethnicity and spirometric restriction should be noted because restriction in this analysis is based on the LLN that in turn is based on race, age, and height [35]. The data collected from 4,320 NHANES participants showed blacks and other race individuals (other than black or white) had odds of restriction on spirometry that were approximately 2–4 times those of whites (blacks, aOR = 1.5 (95% CI: 1.1, 2.0); other race, aOR = 3.7 (95% CI: 1.8, 7.8)) [8]. Similarly, black veterans and veterans of other races/ethnicities in the current study’s analysis had odds of spirometric restriction that were three times the odds of white veterans (black versus white, aOR = 3.04, 95% CI: 1.36, 6.79; other races/ethnicities versus white, aOR = 2.78, 95% CI: 0.96, 8.06). 

This study found a significant association between waist circumference and spirometric restriction, which is consistent with the results from other studies [12,44,45]. This effect has been attributed to chest wall and diaphragm restriction resulting from increases in abdominal mass [44,46]. The relevance of this relates to obesity’s association with increased chronic respiratory and metabolic disease risk and its prevalence in the veteran population—35% of ACC veterans were obese (BMI ≥ 30 kg/m^2^) [26,29], while 41% of approximately 4.5 million male veteran users of VA healthcare were classified as obese [47]. 

There is very limited research on the various types of nonmalignant pulmonary conditions in Vietnam War veterans and very limited use of objective verification measures of both chemical exposure and respiratory status. In an ACC veteran study (*n* = 5,609) published in 2006 [17] on which the current health survey was based, a significant association between self-reported herbicide exposure and physician diagnosed nonmalignant respiratory disease was found (aOR = 1.62, 95% CI: 1.28, 2.05). In that study and in the current study, the veterans’ herbicide exposure self-report provided at separate time periods years after exposure were highly consistent with serum TCDD levels collected in 1999–2000 [17,29,48]. No data on either restrictive disease or any other subcategory of pulmonary disease was obtained in 2006 [17]. The only other study of herbicide exposed veterans with biological measures of exposure, the Air Force Health Study (AFHS) that examined service members who aerially distributed herbicides, did not report results of an examination of restrictive disease [49]. Other large-scale veteran studies, like the Center for Disease Control’s Vietnam Experience Study (VES) [15] conducted in the 1980’s or the Korean Veteran Health Study (KVHS) [27,28], did not examine restrictive pulmonary disease; and in neither of these studies were veteran participants reported to have had chemical operations occupations. To the authors’ knowledge, none of the past studies of Vietnam War veterans, except for the VES, AFHS, and the current ACC study, assessed pulmonary function using spirometry. Furthermore, both the AFHS and the VES reported findings for FEV_1_, FVC, and FEV_1_/FVC without reference to a specific respiratory condition [15,49]. There have also been no studies of Vietnam War veterans with published findings based on full lung function testing involving lung volume and diffusion capacity measurement. The health studies of Australians who served during the Vietnam War were based on self-reports of pulmonary disorders [23,24], whereas the studies of New Zealand Vietnam War veterans cited the use of hospital admission and mortality data [18,19].

Extensive research has been conducted on other populations with potential exposure to herbicides and pesticides, such as agricultural workers [1,2]. Many of these agricultural studies examined chemicals like the nitrogen-containing paraquat (methylated bipyridinium dichloride) and organophosphates (esters of phosphoric acid) [50,51,52,53,54]. These chemicals are not structurally related to the phenoxy herbicides sprayed during the Vietnam War and others, like paraquat, were not used during that conflict [2]. Findings may still inform our results because the exact range of chemical exposures experienced by the ACC or other Vietnam War veterans during the War or at other times in their lives is not known. 

Exposure to paraquat, a chlorinated pyridine derivative, has been studied as a potential contributor to restrictive disease (e.g., pulmonary fibrosis). A significant trend (*p* = 0.015) was found between spirometrically-determined restrictive ventilatory dysfunction and paraquat exposure in South Korean farmers (*n* = 2,805, those with acceptable values on spirometry) [50]. However, in approximately 300 Costa Rican farm workers, no association was found between handlers and nonhandlers of paraquat and restrictive lung disease based on spirometric parameters, total lung capacity (TLC_58_), and single-breath carbon monoxide diffusing capacity (DL_CO_) [51]. The pesticide sprayers (*n* = 89) who were exposed to neonicotinoids, insecticides that are chemically like nicotine and which were handled by 81.5% of these sprayers, were found to have lowered pulmonary volumes (total lung capacity, residual volume, functional residual capacity as determined by the helium dilution technique) and diffusing capacity of the lungs that were indicative of restrictive lung disease [52]. 

A spirometric investigation of the association of organophosphate pesticides and restrictive disease in Indian farmers (*n* = 25) and fishermen (*n* = 22) showed that occupationally exposed farmers had significantly lower (*p* < 0.023) FVC ratios (ratios of observed to predicted values) than controls during in-season periods, and significantly lower FEV_1_ ratios (*p* < 0.05). These findings could not be replicated in fishermen who were environmentally exposed to organophosphate pesticides [53]. Similar findings of the effects of organophosphate and carbamate pesticides were reported more recently in 376 Indian farmers where restrictive deficits using spirometry were noted [54]. 

The technical capabilities of spirometry are a limitation. Spirometry, although deemed an adequate method to identify restriction when FVC is used in conjunction with FEV_1_/FVC [13], is still only a surrogate for gold standard methods that determine total lung capacity [55]. Spirometry does not provide details as to restrictive disease type, so the authors were unable to determine the existence of any forms of interstitial lung disease or clarify any subtype, which depend heavily on specific patterns of chest imaging, clinical history, and measurement of lung volumes and diffusion capacity. Full pulmonary function tests and other more advanced pulmonary assessments were not conducted as part of the original study design because its primary purpose was to evaluate the association between herbicide exposure and hypertension and COPD from data collected through a nationwide health survey of veterans. 

The in-home examination participants were not randomly selected. This may limit the generalizability of results. The study was powered on the expected prevalence of self-reported COPD for the main component of the study, one of two chronic health conditions of primary interest in the original research protocol and thus on detecting significant differences in the prevalence of a COPD diagnosis using non-spirometric methods. It was not powered on detecting differences based on spirometric restriction prevalence, which may explain why no significant difference was found by exposure level despite the magnitude of the difference in prevalence.

Mortality from nonmalignant pulmonary disease or from restrictive lung disorders was not determined as part of the analyses presented here and that could serve to underestimate pulmonary disease prevalence in this population. The last mortality study of the ACC showed a marginally significant increased mortality risk in nonmalignant pulmonary conditions when comparing men who served in Vietnam versus those who served outside Southeast Asia (adjusted relative risk (ARR) = 2.20, 95% CI: 0.99, 4.91). There was a significant increase in COPD mortality risk between the two groups (ARR = 4.82, 95% CI: 1.10, 21.18), while no estimates of mortality risk were provided for those with any restrictive pulmonary disorder. A further subanalysis in the same study examined COPD between Vietnam sprayers versus Vietnam nonsprayers. Although the mortality risk was significantly higher among Vietnam sprayers relative to Vietnam nonsprayers (ARR = 3.55, 95% CI: 0.39, 32.14), the association was questionable due to the very low number of deaths (n = 6) ascertained from 31 March 1973 (the date that marked the end of U.S. combat involvement in the Vietnam War) through 31 December 2005 [25]. 

Future research could approach the examination of restrictive disease using a case-control design in which veterans with spirometric restriction could be compared to veterans with a normal pattern. Because restrictive disease physiology requires disease confirmation by the measurement of lung volumes, the cases and controls would be identified based on comprehensive clinical diagnostic evaluations and matched on specified characteristics. A greater examination of the differences between the cases and controls could then be made by collecting a much broader set of variables related not only to herbicides, but to other exposures and explanatory variables, such as those related to the many determinants of health that are yet unaddressed. 

This study has several strengths: High response rates for each of its three research phases (79.3%, survey; 93.4%, medical records; 63.8%, in-home physical examinations) (Figure 1) [26,29]; herbicide exposure based on self-reports confirmed with blood serum values of TCDD collected in 1999–2000 [29]; and Vietnam War deployment status and other service-related data that were documented and verified using military personnel records. The listing of ACC personnel is comprehensive and the deployed and nondeployed cohorts were developed to optimize comparability on the basic demographic and service-related characteristics. In addition, spirometric restriction prevalence in this cohort for unexposed veterans (9.9%, 95% CI: 5.9, 13.9) compared well to the estimates of the general U.S. population for those 60–79 years of age using 2007–2010 NHANES data where spirometric restriction was defined as FEV_1_/FVC ≥ 0.70 and FVC < 80% predicted (mean (± standard error) = 10.6 (± 0.9)) [56]. This study also used statistical procedures that strived to reduce overfitting of the regression models and control for confounding. 

## 5. Conclusions

This subanalysis on spirometric restriction derived from this veteran health study has several strengths which support the need for continued research on interstitial pulmonary disease for veterans, not only of the Vietnam War, but for veterans of other military conflicts. These results are important because there are no published findings on restrictive pulmonary disease in Vietnam War veterans and because herbicide and other chemical exposures, hazards that are frequently characteristic of military service, are implicated in its etiology. Further work should include full pulmonary function testing, the application of other types of research designs where exposure and outcome associations could be more clearly delineated, and the incorporation of a wider set of explanatory variables in analysis, such as other social determinants of health.

## Figures and Tables

**Table 1:** The characteristics of U.S. Army Chemical Corps spirometry participants, stratified by herbicide exposure.

Characteristic ^a^	All (*n* = 403)	Exposed (Herbicide Sprayers) (*n* = 191, 47.39%)	Unexposed (Herbicide Nonsprayers) (*n* = 212, 52.61%)	*p* Value
Age (yrs), at time of study *n (%)*				
59–64	121 (30.0)	49 (25.7)	72 (34.0)	0.076
65–69	218 (54.1)	105 (55.0)	113 (53.3)	
70+	64 (15.9)	37 (19.4)	27 (12.7)	
Age (yrs) *mean (± SD)*	66.4 (± 3.85)	66.8 (± 3.85)	66.0 (± 3.82)	0.053 *
Race/ethnicity *n (%)*				
White	301 (74.7)	133 (69.6)	168 (79.3)	0.078
Black	65 (16.1)	38 (19.9)	27 (12.7)	
Other nonwhite	37 (9.2)	20 (10.5)	17 (8.0)	
Vietnam service status *n (%)*				<0.0001 *
In-theater (Vietnam)	272 (67.5)	162 (84.8)	110 (51.9)	
Outside SEA (non-Vietnam)	131 (32.5)	29 (15.2)	102 (48.1)	
Officer status *n (%)*				0.23
Yes	62 (15.4)	25 (13.1)	37 (17.5)	
No (enlisted)	341 (84.6)	166 (86.9)	175 (82.6)	
Military service duration (mos) *n (%)*				0.017 *
18–23	96 (23.8)	41 (21.5)	55 (25.9)	
24–36	218 (54.1)	96 (50.3)	122 (57.6)	
> 36	89 (22.1)	54 (28.3)	35 (16.5)	
Cigarette smoking *n (%)*				0.14
Current	55 (13.9)	29 (15.5)	26 (12.4)	
Former	208 (52.4)	104 (55.6)	104 (49.5)	
Nonsmoker	134 (33.8)	54 (28.9)	80 (38.1)	
Missing	6			
BMI (kg/m^2^) *n (%)*				0.58
< 24.9	66 (16.4)	34 (17.8)	32 (15.1)	
25.0–29.9	151 (37.5)	67 (35.1)	84 (39.6)	
≥ 30	186 (46.2)	90 (47.1)	96 (45.3)	
Waist: hip ratio *n (%)*				0.61
≥ 0.90	341 (84.8)	163 (85.8)	178 (84.0)	
< 0.90	61 (15.2)	27 (14.2)	34 (16.0)	
Missing	1			
Waist circumference (cm) *n (%)*				0.63
≥ 102	189 (46.9)	92 (48.7)	97 (45.8)	
< 102	214 (53.1)	99 (51.8)	115 (54.3)	
Height (cm) *mean (± SD*)	175.8 (± 7.52)	175.8 (± 7.47)	175.9 (± 7.58)	0.89
COPD *n (%)*				<0.0001 *
Yes	65 (16.4)	47 (25.3)	18 (8.5)	
No	332 (83.6)	139 (74.7)	193 (91.5)	
Missing	6			
Hypertension *n (%)*				0.011 *
Yes	289 (71.9)	148 (77.9)	141 (66.5)	
No	113 (28.1)	42 (22.1)	71 (33.5)	
Missing	1			
Diabetes *n (%)*				0.056
Yes	75 (18.6)	43 (22.5)	32 (15.1)	
No	328 (81.4)	148 (77.5)	180 (84.9)	

BMI, body mass index; cm, centimeters; COPD, chronic obstructive pulmonary disease; kg, kilograms; m, meters; mos, months; SD, standard deviation; SEA, Southeast Asia; yrs, years. ^a^ Age, race/ethnicity, BMI, waist: hip ratio, waist circumference data were obtained from the physical examination and spirometry session; military service data were obtained from military personnel records; Herbicide sprayer status, cigarette use, COPD, and hypertension data were obtained from the survey—COPD and hypertension were based on physician diagnosis; diabetes data were obtained from medical records. * Statistically significant (*p* ≤ 0.05).

**Table 2:** The spirometry characteristics of U.S. Army Chemical Corps veterans, stratified by herbicide exposure.

Spirometry Parameters	All Veterans (*n* = 403)	Exposed (Herbicide Sprayers) (*n* = 191)	Unexposed (Herbicide Nonsprayers) (*n* = 212)	*p* Value
FEV_1_ *mean (± SD)*	2.89 (± 0.66)	2.85 (± 0.65)	2.93 (± 0.66)	0.23
FVC *mean (± SD)*	3.97 (± 0.81)	3.90 (± 0.78)	4.04 (± 0.83)	0.082
FEV_1_/FVC *mean (± SD)*	72.78 (± 8.33)	73.01 (± 8.47)	72.57 (± 8.22)	0.60
% of predicted, FEV_1_ *mean (± SD)*	89.96 (± 18.94)	89.55 (± 19.63)	90.32 (± 18.33)	0.69
% of predicted, FVC *mean (± SD)*	92.24 (± 16.42)	91.37 (± 16.32)	93.03 (± 16.51)	0.31
Prevalence of abnormality				
Spirometric restriction ^a^ % *(95% CI)*	12.7 (9.8, 16.3)	15.7 (10.6, 20.9)	9.91 (5.9, 13.9)	0.081
Mixed process of spirometric restriction and obstruction ^b^ *% (95% CI)*	4.5 (2.8, 7.0)	4.7 (1.7, 7.7)	4.3 (1.5, 7.0)	0.82

CI, confidence interval; FEV_1_, forced expiratory volume in 1 second; FVC, forced vital capacity; LLN, lower limit of normal (lower fifth percentile of a large healthy reference group); SD, standard deviation. ^a^ Spirometric restriction defined as FEV_1_/FVC ≥ LLN & FVC < LLN. ^b^ Mixed process of spirometric restriction and obstruction defined as FEV_1_/FVC < LLN & FVC < LLN.

**Table 3:** The adjusted odds ratios and 95% confidence intervals for the association between spirometric restriction with herbicide exposure and other independent variables ^a^.

Independent Variables ^b^	aOR (95% CI)
Spray status (ref = nonsprayer)	1.64 (0.82, 3.29)
Age, at time of study, 65–69 yrs (ref =59–64 yrs)	1.16 (0.51, 2.61)
Age, 70+ yrs	2.03 (0.65, 6.37)
Race/ethnicity, black (ref = white)	3.04 (1.36, 6.79) *
Race/ethnicity, other nonwhite	2.78 (0.96, 8.06)
Vietnam (ref = non-Vietnam)	0.88 (0.40, 1.95)
Rank (ref = Officer)	0.57 (0.21, 1.52)
Military duration, 24–36 mos (ref =18–23 mos)	0.70 (0.33, 1.50)
Military duration, >36 mos	0.68 (0.25, 1.90)
Cigarette smoker, current (ref = nonsmoker)	1.43 (0.50, 4.14)
Cigarette smoker, former	1.30 (0.63, 2.70)
Waist circumference (≥102 cm versus <102 cm (ref))	2.46 (1.25, 4.85) *
COPD (ref = no COPD)	0.83 (0.36, 1.94)
Height, cm	1.04 (1.00, 1.09)

aOR, adjusted odds ratio; CI, confidence interval; cm, centimeters; COPD, chronic obstructive pulmonary disease; mos, months; ref, referent category; yrs, years. ^a^ The likelihood ratio chi-square for testing the global null hypothesis (*β* = 0) was 27.74, 14 df (*p* = 0.015). Analysis based on 403 observations (*n* = 51 veterans with spirometric restriction). Estimates of associations are derived from a single multivariable model. ^b^ Herbicide exposure, cigarette use, COPD data were obtained from the survey—COPD was based on physician diagnosis; age, race/ethnicity, waist circumference, height data were obtained during the physical examination and spirometry session; military service data were obtained from military personnel records. * 95% CI does not contain 1.0.
